# Histone deacetylase inhibitors induce expression of chromosomally tagged variant-specific surface protein genes in *Giardia lamblia*

**DOI:** 10.1186/s13104-020-04995-6

**Published:** 2020-03-12

**Authors:** Daniel Roberto Orozco, Srinivas Garlapati

**Affiliations:** Biology Program, School of Sciences, University of Louisiana at Monroe, Chemistry and Natural Sciences Building, 700 University Avenue, LA Monroe, 71209 USA

**Keywords:** *Giardia lamblia*, Variant specific surface protein, Endogenous tagging, Histone deacetylase inhibitors, Antigenic variation, Histone deacetylase inhibitors

## Abstract

**Objective:**

RNA interference and miRNA mediated mechanisms have been proposed to explain the expression of a specific variant of VSP at a time on the surface of *Giardia lamblia*. Recently, epigenetic mechanisms involving histone acetylations have been proposed to explain the process of *vsp* gene switching in *Giardia lamblia*. However, due to the limited availability of specific antibodies for all the *vsp* variants present in the genome, it was difficult to monitor *vsp* gene switching. In this study, we have used an endogenous tagging method to tag specific *vsp* genes *vsp1267* and *vsp9B10A* with a sequence encoding hemagglutinin (HA) epitope at the 3′end of the coding sequences without altering the 5′ upstream elements. With this method, we have monitored the expression of the tagged *vsp* genes in cells treated with histone deacetylase inhibitors using RT-PCR.

**Results:**

Our results show that *vsp1267*-*3XHA* can be induced by treatment with sodium 4-phenylbutyrate, M344 and splitomicin but not by apicidin and Trichostatin A, while *vsp9B10A*-*3XHA* expression can be induced by Trichostatin A and splitomicin but not by sodium 4-phenylbutyrate, M344 and apicidin. The induced expression of these variants was not due to growth inhibition. These results support the role of histone acetylations in vsp expression.

## Introduction

*Giardia lamblia* antigenic variation involves the expression of a single variant-specific surface protein (VSP) on the trophozoite surface from a library of approximately 200 distinct *vsp* genes [1]. VSPs cover the entire surface of *Giardia* trophozoites, including the flagella and ventral disk [2]. The dense transmembrane regions of VSPs are speculated to provide a protective barrier to prevent the host immune system from accessing the trophozoite plasma membrane [3]. Although RNAi and miRNA mechanisms that target the coding sequences of *vsp* transcripts explain how one VSP is expressed at a time [4, 5], they do not explain how VSP switching occurs. Epigenetic mechanisms have been proposed to explain the VSP switching in *Giardia* [3, 6, 7]. Recently, it was demonstrated that histone deacetylase inhibitor (HDACi) Trichostatin A increases the rate of VSP switching after 5 days of treatment, while sodium butyrate and nicotinamide had minor effects on switching rates [6]. The increase in switching rate was attributed to the increase in the association of H3K9ac, H4K8ac, and H4K16ac with the 5′ upstream sequences of *vsp*1267, the specific *vsp* used in the study. Also, the genes that not are expressed (*vsp910B* and *vspA6*) showed a significant decrease in the acetylation of histones associated with the 5′ upstream sequences [6]. However, it was not possible to detect the new VSPs expressed after switching due to a limitation in the availability of monoclonal antibodies for all the 200 possible variants of VSPs [6].

In this study, we have monitored the expression of chromosomally-tagged *vsp* genes *vsp1267* and *vsp9B10A* in separate cell lines that were treated with histone deacetylase inhibitors. Due to a high degree of sequence similarity of VSPs, monitoring the expression of a specific *vsp* gene is difficult as it requires monoclonal antibodies that recognize it. However, by tagging a specific *vsp* gene, it is easy to detect its expression by RT-PCR. In order to ensure our chromosomally integrated construct is reflective of an endogenous *vsp*, the 5′ end of the *vsp* gene was unaltered, which is where epigenetic mechanisms generally operate. Our results show that treatment with HDAC inhibitors resulted in the expression of the tagged *vsp* gene more often than compared to untreated controls.

## Main text

### Methods

#### VSP cloning

**Fig. 1:**
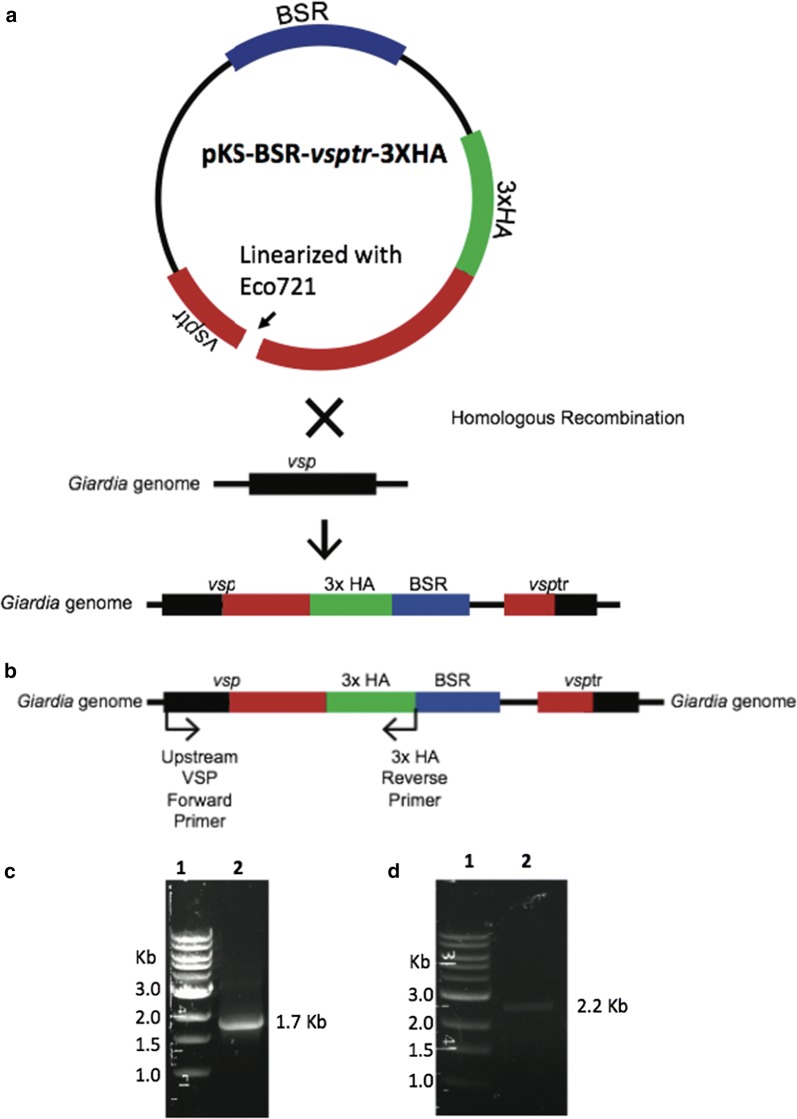
Tagging of a chromosomal copy of *vsp* gene with 3XHA epitope using endogenous tagging method. **a** The plasmid construct pKS-BSR-*vsptr*-3XHA containing blasticidin resistance gene (BSR, highlighted in blue), truncated *vsp* gene (*vsptr*, highlighted in red), and three copies of hemagglutinin epitope (3XHA, highlighted in green) were linearized with the restriction enzyme *Eco721* and then integrated into *Giardia* genome by homologous recombination. **b** The forward and reverse primers used for amplifying the full length *vsp* gene tagged with 3XHA are indicated by arrows. **c** Agarose gel electrophoresis of DNA size maker (lane 1) and the amplicons representing the full length vsp1267 gene tagged with 3XHA in Gl*vsp*1267-3XHA cell lines (lane 2). **d** Agarose gel electrophoresis of DNA size maker (lane 1) and the amplicons representing the full length vsp910BA gene tagged with 3XHA in Gl*vsp*9B10A-3XHA cell lines (lane 2)

The truncated *vsp1267* gene (GL50803_112208) lacking the coding sequences for the first 20 amino acids (Fig. 1a) was amplified using the primers 5′-CAC*gcggccgc*TGGAAATAGTTGTGAAGCTGG-3′ (forward) and 5′-CAC*ctcgag*CGCCTTCCCCCTGCATATG-3′ (reverse) that contain recognition sequences for restriction enzymes *Not I* and *Xho I*, respectively. Similarly, truncated *vsp9B10A* gene (GL50803_101074) lacking the coding sequences for the first 20 amino acids (Fig. 1a) was amplified using the primers 5′-CACgcggccgcAACAGAGCGCGCGCAAGAAGCTC-3′ (forward) and 5′-GTGctcgagCGCCTTGCCTCTGCACATAAAC-3′ (reverse) containing sites for enzymes *NotI* and *XhoI*, respectively. The amplified genes were cloned into pGEM-T easy vectors (Promega, Madison WI) and the recombinant plasmids were sequenced. The truncated *vsp* genes (*vsp1267tr* and *vsp9B10Atr*) from pGEM-T easy vectors were then cloned into pKS-BSR-3XHA vector [8] upstream of a 3X HA sequence to generate pKS-BSR-vsp1267tr-3XHA and pKS-BSR-vsp9B10Atr-3XHA.

#### Transfection

Recombinant plasmids pKS-vsp1267-3XHA and pKS-vsp9B10A-3XHA were linearized using *Eco721* restriction enzyme, which cuts once in the coding sequences of truncated *vsp* genes. The digested plasmids were then transfected into *Giardia lamblia* WB trophozoites and selected for blastidicin resistance as described previously [8]. To confirm the chromosomal integration and 3XHA tagging of a copy of *vsp* gene, a forward primer corresponding to the 5′end of the coding sequence of the *vsp* gene (*vsp1267* primer 5′-ATGTTGTTGATAGCCTTCTATC-3′; *vsp9B10A* primer 5′-GTGCATATGACTGCCAAACATTGCCCGATTGATAGATTG-3′) and a reverse primer (5′-TCAGGATCCAGCGTAATCTGGTAC-3′) corresponding to the 3XHA tag sequence from the plasmid vector were used to amplify the endogenously tagged *vsp* genes.

#### Vsp gene expression

Total RNA was extracted from untreated and HDAC inhibitor treated cells using TRIzol reagent by following the manufacturer’s instructions (Invitrogen). RNA samples were treated with DNAse I (New England Biolabs) for 30 min at 37 °C to remove DNA contamination. One µg of treated RNA was used for cDNA synthesis using OneTaq RT-PCR kit (New England Biolabs) by following the manufacturer’s instructions. To amplify full-length transcripts with 3XHA tag, a forward primer that encompasses the entire length of the coding sequence and a reverse primer that corresponds to 3XHA were used. Coding sequences of the full length GleIF4A (GL50803_10255) transcripts were used as an internal control.

#### Effect of HDACi on the growth of Giardia

*Giardia* cell lines were grown to mid-late log phase of growth and were sub-cultured to contain approximately 10^5^ cells/mL and treated with HDAC inhibitors apicidin, trichostatin A (TSA), sodium 4-phenylbutyrate (NaPB), M344 and splitomicin (Sigma-Aldrich) at a final concentration of 2µM. Trophozoites were incubated at 37 °C and the growth of parasites were monitored by counting the cells using a hemocytometer after 24 and 48 h post-treatment.

### Results

#### Endogenous tagging

The plasmid constructs pKS-BSR-vsp1267tr-3XHA and pKS-BSR-9B10Atr-3XHA were linearized with *Eco721* and transfected into *Giardia* cells (Fig. 1a). To confirm the integration of the truncated and tagged version of the *vsp* gene, primers encompassing the entire coding sequence starting from the initiation codon to the stop codon located after the triple HA tag were used (Fig. 1b). Amplification of full length HA tagged *vsp*1267 of size 1.7 Kb (Fig. 1c lane 2) and full length HA tagged *vsp*9B10 of size 2.2 Kb (Fig. 1d, lane 2), indicated proper integration of the 3XHA tagged gene into the chromosome.

#### HDACi induce transcription of endogenously tagged vsp genes

**Fig. 2:**
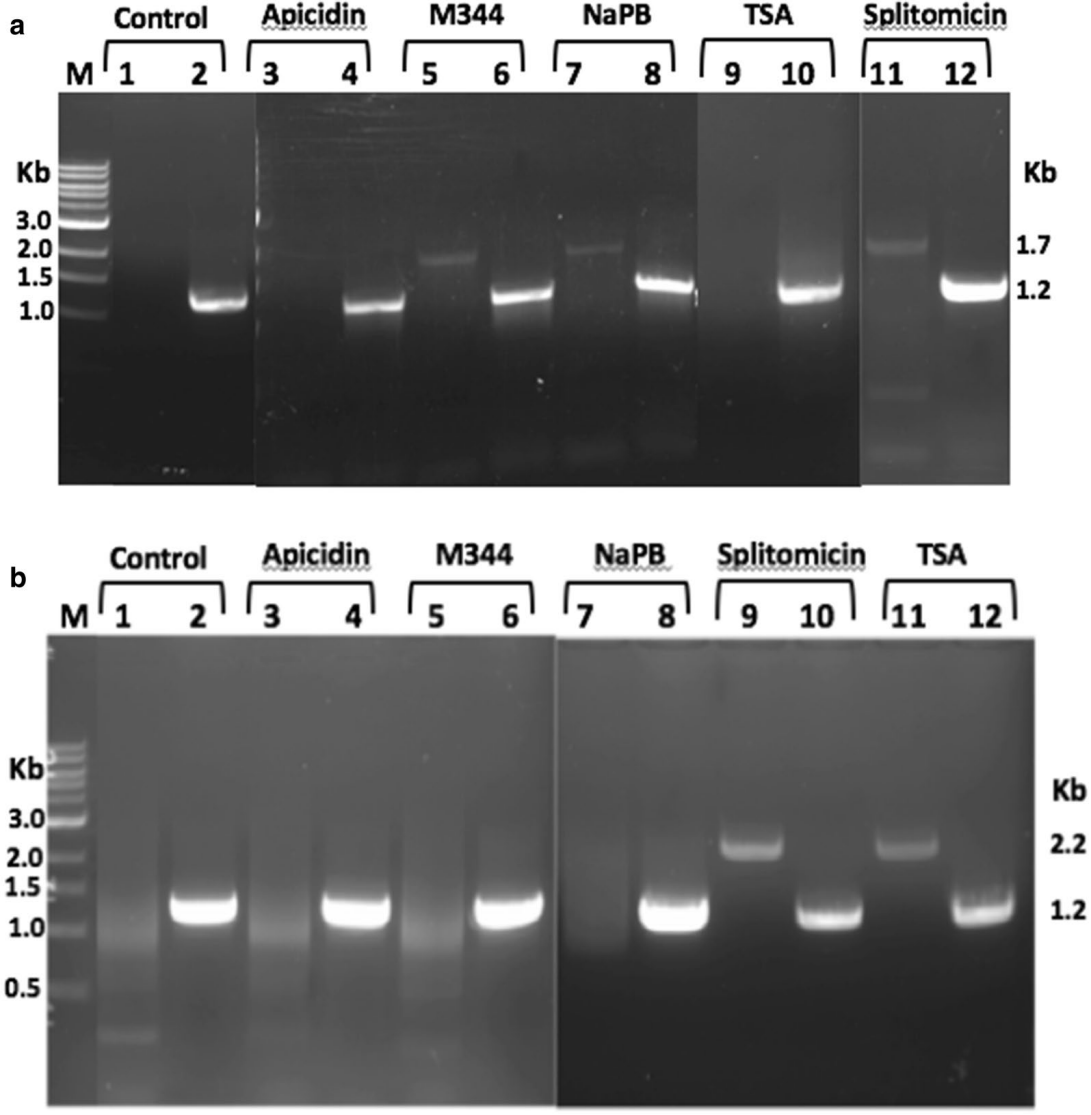
Induction of *vsp* gene expression in *Giardia* trophozoites treated with Histone deacetylase inhibitors. **a** RT-PCR analysis of full length vsp1267-3XHA transcripts in untreated Glvsp1267-3XHA cell lines (lane 1), in cell lines treated with Apcidin (lane 3), M344 (lane 5), NaBP (lane 7), splitomicin (lane 9) and TSA (lane 11). RT-PCR of full length transcripts of GleIF4A (lanes, 2, 4, 6, 8, 10, and 12) was used as an internal control. **b** RT-PCR analysis of full length vsp9B10A-3XHA transcripts in untreated Glvsp9B10A-3XHA cell lines (lane 1), in cell lines treated with Apcidin (lane 3), M344 (lane 5), NaBP (lane 7), splitomicin (lane 9) and TSA (lane 11). RT-PCR of full length transcripts of GleIF4A (lanes, 2, 4, 6, 8, 10, and 12) was used as an internal control

To test the hypothesis that inhibition of histone deacetylase activity leads to the expression of *vsp* genes, trophozoites were incubated with 2 µM concentrations of HDAC inhibitors for 24 h. Total RNA was extracted from *Giardia* cell lines Glvsp1267-3HA and Glvsp9B10A-3XHA after 24 h post-treatment. Reverse transcription polymerase chain reaction (RT-PCR) was performed to detect for HA-tagged *vsp* gene expression using a full-length *vsp* primer and a 3XHA primer (Fig. 1b). Additionally, *Giardia lamblia* eukaryotic translation initiation factor 4A (*GleIF4A*) was used as an internal control. A full length amplicon of size 1.7 Kb was detected in Glvsp1267-3XHA cell lines that were treated with M344, NaPB and splitomicin but not in the untreated control (Fig. 2a, lanes 5, 7, 11). For M344 and NaPB treatments, full length amplicon of vsp1267-3XHA was detected in 2 out of 3 separate experiments performed on different days. In splitomicin treated cell lines, 8 out of 13 independent experiments detected full length amplicon. In the experiments where the full length amplicons were not detected when treated with M344, NaPB or splitomicin, a smear of amplicons ranging from 0.5 to 1 Kb were observed (data not shown). These partial amplicons could represent the intermediates of degraded *vsp1267* transcripts. Interestingly, no full length amplicons were detected when the cells were treated with apicidin or TSA (Fig. 2a, lanes 3 and 9) and the results were consistent in 7 independent treatments performed on different days. These results agree with the previous reports that showed a decrease in the expression of *vsp1267* upon treatment with TSA [4] or no change in expression when treated with apicidin [9]. Both untreated control and HDAC inhibitor treated cells showed a full-length GleIF4A amplicon of 1.2 kb (Fig. 2a, lanes, 2,4, 6, 8, 10 and 12).

In contrast to vsp1267-3XHA, full length vsp9B10A-3XHA amplicon of 2.2 Kb was detected in Glvsp9B10A cell lines that were treated with splitomycin or trichostatin A (Fig. 2b, lanes 9 and 11, respectively) but not in cell lines treated with apicidin, M344 or NaPB (Fig. 2b, lanes 3, 5, and 7, respectively). However, a smear of amplicons ranging from 0.5 to 1.5 Kb was observed in untreated control and in Apicidin, M344 or NaPB treated cells but not in cells treated with splitomicin or TSA. These amplicons may represent the intermediates of transcripts degraded by RNAi and/or miRNA mediated mechanisms [4, 5]. Both in untreated control and treated cell lines, the expression of the internal control gene GleIF4A was detected (Fig. 2b, lanes, 2, 4, 6, 8, 10 and 12).

#### Effect of HDACi on parasite growth

**Fig. 3:**
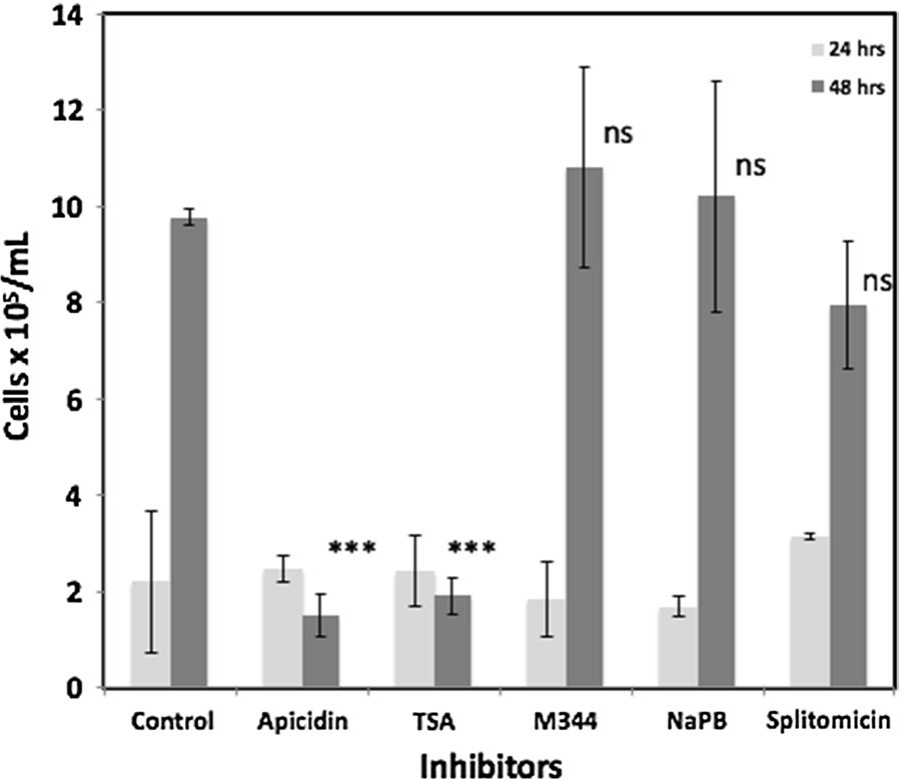
Effect of Histone deacetylase inhibitors on the growth of *Giardia* cell lines Glvsp1267-3XHA. The parasite cultures in triplicates were incubated with various HDAC inhibitors at a final concentration of 2µM for 48 h. The cell numbers were counted after 24 and 48 h of incubation using a hemocytometer. The averages of triplicates were plotted with error bars indicating standard deviation. The significance of the difference between the control and the treated cells was calculated using the pairwise t test

Replicating trophozoites of Glvsp1267-3XHA cell lines were treated with 2 µM of HDAC inhibitors for up to 48 h. After 24 h, no significant growth inhibition was observed in the cell lines treated with all the inhibitors when compared to control (Fig. 3). However, after 48 h of treatment, Apicidin inhibited the parasite growth by 87% (P < 0.001%), while Trichostatin A inhibited the growth by 82.2% (P < 0.001%) when compared to the untreated controls. However, there was no significant decrease in the growth of the parasites when treated with M344, NaPB, or splitomycin, when compared to the untreated controls (Fig. 3). These results suggest that the changes in the expression of vsp1267-3XHA (Fig. 2a) observed after 24 h of HDAC inhibitor treatment were not due to cell toxicity.

### Discussion

In this study, we demonstrated that tagging a chromosomal copy of a *vsp* gene can be used to monitor its expression in the presence of histone deacetylase inhibitors. Since the genes are tagged with 3XHA epitope at the 3′end, the 5′ end is not disrupted [8]. Thus, it maintains the native chromatin environment at the promoter region for the epigenetic mechanisms to operate. Also, tagging the 3′end does not necessarily interfere with the RNAi and miRNA mediated silencing mechanisms as they target the coding sequences of *vsp* mRNA in *Giardia* [4, 5].

Due to the unavailability of monoclonal antibodies, we were not able to confirm the variant of *vsp* that is being expressed by *Giardia* WB cells before generating 3XHA tagged cell lines. Since we did not detect the expression of the tagged *vsps* (*vsp*1267 and *vsp*9B10) in untreated controls, we assume that these cell lines (Gl*vsp*1267-3XHA and Gl*vsp*9B10-3XHA) are probably expressing a different variant of *vsp* at the time of treatment with HDAC inhibitors. Although we were able to detect the expression of the tagged *vsp* transcripts in treated cells, but not an untagged *vsp*, we cannot yet dismiss the possibility that these cells may have switched the *vsp* gene from an unknown variant to the tagged *vsp*.

In our experiments, we have detected the expression of tagged *vsps* in cell lines treated with splitomicin more often than treated with TSA, Apicidin, M344, and NaPB, when compared to untreated controls. Apicidin [10], TSA [11], NaPB [12], and M344 [13] are known to inhibit NAD + independent histone deacetylases that belong to class I HDACs. A homologue of class I HDAC has been identified in the *Giardia* genome database and previous reports have indicated that this enzyme can be inhibited by TSA, Apicidin, and NaPB [6, 8]. In agreement with the previous reports [6, 8], we have observed growth inhibition of the parasites within 48 h of treatment with TSA and Apicidin. Although M344 and NaPB are known to target the same HDAC I enzyme, they did not inhibit parasite growth even after 48 h of treatment. These results suggest that growth inhibition by TSA and Apicidin could be due to the inhibition of other essential cellular processes in addition to the inhibition of HDAC I enzyme [8]. Previous reports have indicated that treatment with TSA leads to an increase in the association of H3K9ac, H4K8ac and H4K16ac with the 5′ upstream sequences of expressed *vsps* [6], as HDAC I enzyme is known to target these acetylated histones. Based on these observations, we speculate that induction of *vsp1267*-*3HA* with M344 and NaPB, and *vsp9B10A*-*3XHA* with TSA, could be due to hyperacetylaton of these histones associated with the 5′upstream elements.

Although *vsp1267*-*3XHA* and *vsp9B10A*-*3XHA* respond similarly to splitomicin [14] (inhibitor of Class III HDACs) treatment, they differ in their response to NaPB, M344 and TSA (inhibitors of Class I HDACs) treatments. It has been demonstrated that cancer cells display a differential response to apicidin, depsipeptide and TSA, and these differences in response were attributed to inhibitors targeting protein factors other than inhibiting HDAC activity in the cells, such as lowering global levels of histone methylation [15]. Therefore, it is likely that HDAC inhibitors could be targeting other proteins involved in gene expression. Alternatively, the chromatin environment at the promoter elements of vsp1267-3XHA and vsp9B10A-3XHA may be different, resulting in differences in the responses to various HDAC inhibitors.

## Limitations

Although tagging the 3′end allowed us to qualitatively monitor the full-length transcripts using RT-PCR, it is not ideal for accurately estimating the transcript levels using qPCR as it requires amplification of small regions of ~ 200 nucleotides in order to accurately estimate transcript levels [16]. Due to RNAi and miRNA mechanisms, short mRNA intermediates are generated and these intermediates could be potentially amplified by RT-qPCR.

## Data Availability

Not applicable.

## Abbreviations

HA: Hemagglutinin
NaPB: Sodium phenylbutyrate
TSA: Trichostatin A
HDAC: Histone deacetylase
HDACi: Histone deacetylase inhibitors
